# Microfluidic Technologies for cfDNA Isolation and Analysis

**DOI:** 10.3390/mi10100672

**Published:** 2019-10-03

**Authors:** Zheyun Xu, Yi Qiao, Jing Tu

**Affiliations:** State Key Laboratory of Bioelectronics, School of Biological Science and Medical Engineering, Southeast University, Nanjing 210096, China; xuzheyun95@163.com (Z.X.); qiaoyilll@163.com (Y.Q.)

**Keywords:** microfluidics, LOC, cfDNA isolation, cfDNA analysis

## Abstract

Cell-free DNA (cfDNA), which promotes precision oncology, has received extensive concern because of its abilities to inform genomic mutations, tumor burden and drug resistance. The absolute quantification of cfDNA concentration has been proved as an independent prognostic biomarker of overall survival. However, the properties of low abundance and high fragmentation hinder the isolation and further analysis of cfDNA. Microfluidic technologies and lab-on-a-chip (LOC) devices provide an opportunity to deal with cfDNA sample at a micrometer scale, which reduces required sample volume and makes rapid isolation possible. Microfluidic platform also allow for high degree of automation and high-throughput screening without liquid transfer, where rapid and precise examination and quantification could be performed at the same time. Microfluidic technologies applied in cfDNA isolation and analysis are limited and remains to be further explored. This paper reviewed the existing and potential applications of microfluidic technologies in collection and enrichment of cfDNA, quantification, mutation detection and sequencing library construction, followed by discussion of future perspectives.

## 1. Introduction

Cell-free DNA (cfDNA), which is known as degraded DNA released to blood, urine or other body fluids, has been proved to be great significance in clinical diagnosis. cfDNA applied to clinical research mainly focuses on the following three parts: Non-Invasive Prenatal Testing (NIPT), organ transplantation monitoring and cancer diagnosis and treatment. The discovery of fetus-derived cfDNA in maternal plasma and serum brought about the most successful application. It was reported that fetus-derived Y-chromosome sequences could be detected in 80% of women bearing male fetuses using only 10 μL plasma samples, which opened a new door to NIPT [1]. Later, a comparison of NIPT using cfDNA and the standard screening for common autosomal aneuploidies detection was carried out. The results showed that cfDNA-based NIPT had significantly lower false-positive rate compared with the conventional screening (for trisomy 21: 0.3% vs 3.6%, P < 0.001; for trisomy 18: 0.2% vs. 0.6%, P = 0.03), where higher overall negative and positive predictive value were also achieved. Another important application for cfDNA is to be a universal biomarker for allograft rejection in solid organ transplantation. Since patients suffering from allograft rejection present a remarkable increase (>5-fold compared to patients without complications) in graft-derived cfDNA (GcfDNA) levels, the quantification of GcfDNA could detect allograft rejection at an early stage to prevent full-blown rejection [2]. 

As for applications in cancer diagnosis, cfDNA in plasma of cancer patients is a mixture of both normal and tumor DNA. It is generally accepted that cfDNA is released from healthy issues, tumor cells or hematopoietic cells into the blood stream due to necrosis or apoptosis mechanism [3]. Such property as a “mixture” makes cfDNA to be a biomarker for cancer. Because of the clinical diversity of tumor, traditional medical treatment is hard to meet the demand for medical care, which highly requires the development of precision oncology. Precision oncology is based on detection of specific genomic alterations and monitoring evolutionary mutation during treatment to identify potential resistance mechanisms [3]. Since cfDNA was first discovered by Mandel and Metais in 1948 [4], functions of tumor-derived cfDNA have been confirmed in informing genomic mutation [5,6], tumor burden [7,8] and drug resistance [9,10]. cfDNA, as an important tool for liquid biopsy, has an advantage over traditional methods for tumor sampling because of its low invasion [11]. Thus, sampling could be conducted at regular intervals for timely feedback of patients. cfDNA also provides more information than tumor tissue, which is limited by spatial and temporal heterogeneity, and finally promotes precision oncology.

However, there are numerous challenges in cfDNA detection, which hinders its further application for “liquid biopsy”. One problem proposed is the low absolute levels of cfDNA. As it was reported by Stemmer and colleagues, the concentration of cfDNA in healthy subjects was 3–22 ng/mL and varied with the isolation method applied [12]. Later Adalsteinsson et al. obtained the similar results [13]. Although the cfDNA concentration in blood of cancer patients is much higher than in healthy subjects, it is still below 100 ng/mL in most cancer patients [3,14]. Such low abundance of cfDNA makes it greatly significant for cfDNA analysis to reduce sample loss during collection and processing. At the same time, improving detection sensitivity and lowering detection limit, which benefits the early detection of cancer, is another direction of efforts for wider application of cfDNA. Another problem we are faced with is the high fragmentation (about 167 bp) of cfDNA [15]. The level of cfDNA has important clinical value as the absolute quantification of total cfDNA is an independent prognostic biomarker of overall survival [16,17], which indicates that patients with higher cfDNA concentration present greater death risk compared to those with low level of cfDNA. This sets higher requirements for precise quantification of cfDNA which may be limited by the high fragmentation property of cfDNA. Besides problems brought by the properties of cfDNA, the time-consuming traditional isolation methods also hamper the point-of-care testing (POCT) of cfDNA, which makes cfDNA less appealing.

**Figure 1:**
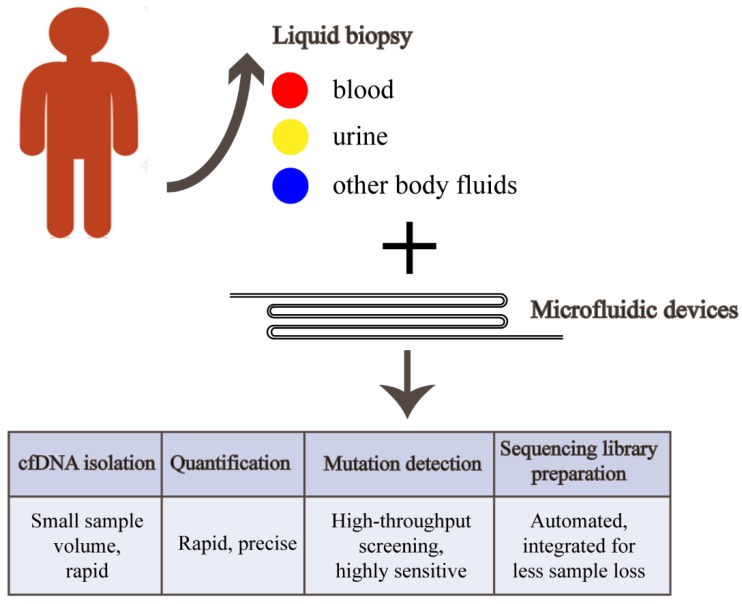
Main advantages that microfluidic devices can bring towards liquid biopsy. cfDNA can be isolated from blood, urine and other body fluids for quantification, mutation detection and next-generation sequencing using microfluidic ways.

About thirty years ago, the concept of miniaturized chemical analysis systems was introduced [18] and has been developed to be new technologies such as “microfluidic” and “lab-on-a–chip (LOC)” devices (here the two terminologies are replaceable) over the two decades [19]. Microfluidic technologies provide an opportunity to manipulate fluid at a micrometer scale [20], making detection at single molecule level possible. Microfluidic devices are suitable for handling micro samples [21,22], which fit the properties of cfDNA and could effectively reduce required sample volume [23]. Furthermore, high degree of automation and high-throughput screening can be performed on microfluidic platform, allowing examination of a large number of patient samples within a short time (Figure 1). LOC devices have been used in many clinical aspects including POCT and research related to genome and protein [24]. However, as a promising method, microfluidic technologies applied to cfDNA research are limited and need to be further explored. This paper reviewed the existing and potential microfluidic applications in collection and enrichment of cfDNA, quantification, mutation detection and sequencing library construction.

## 2. Collection and Enrichment of cfDNA

### 2.1. Traditional Approaches for cfDNA Collection

Traditional approaches for cfDNA collection from plasma, serum and urine rely on commercial kits [25]. Most of the available kits are designed based on the fact that special silicon matrix materials adsorb DNA efficiently and specifically in a certain high-salt buffer system. Other methods like employing the ion-exchange binding of DNA or using organic reagent (phenol/chloroform) for protein denaturation and DNA separation are also commonly used in daily experiments [26]. However, these methods have been proved to be costly, time consuming, with a requirement of large sample volumes [27]. Thus, there is a need to find a more efficient and subtle way for cfDNA collection, probably using microfluidic technologies that can be further developed as a clinical examination standard.

### 2.2. Microfluidic Devices for cfDNA Isolation

Microfluidic techniques in cfDNA capture are limited and have not been widely explored, probably because the relevance between cfDNA and cancer has not been discovered until more recent years. Here we reviewed cfDNA isolation microdevices reported and previously reported microfluidic devices for DNA collection, which are potential ways to be used in cfDNA research.

Microfluidic devices for DNA isolation are grouped into two categories [28]. One is solid phase isolation, which is based on the use of a functionalized surface or immobilized beads for DNA capture. The other is called liquid phase isolation, which uses chemical reagents or relies on electrophoresis (EP)/dielectrophoresis (DEP) to force negatively charged DNA to migrate.

#### 2.2.1. Solid Phase Isolation

Methods of solid phase isolation usually take advantages of silica as solid absorbent [29]. The non-specific absorption between DNA and silica has been applied to DNA isolation for decades. DNA isolation in microchip was first put forward by Christel et al., by means of using a silicon microdevice with pillar structures to get high surface-area-to-volume ratios [30]. Cady et al. used the similar way to create a silicon channel which contained square pillars deposited with a layer of silica, achieving an increase of 6-fold compared to microchannel alone [31]. However, the device fabrication process involved reactive ion etching for pillars creation, which made such device fabrication costly and less efficient. 

Then sol-gel immobilized silica particles in microfluidic devices were developed to reduce the complexity of device preparation procedure. A sol-gel is a colloidal suspension which can transform from a liquid to a solid phase with acid-base catalyst. The silica bead/sol-gel hybrid microfluidic devices were fabricated with a process in which the silica beads were packed into microchannel at first and then immobilized with sol-gel [32]. Later, Wu et al. improved the method by adding poly-(ethylene glycol) which generated pores in the silica matrix and provided large surface area [33]. Absorbed DNA can be purified by wash buffer to remove impurities that are possibly absorbed onto the silica, and finally eluted in a low-salt buffer system for downstream analysis. The efficiencies for extraction of human genomic DNA from blood were ~70%, which was comparable to commercial isolation methods [34]. The same approach has been developed in many respects with an example that Park et al. presented spatially controlled silica coating with sol-gel [35]. 

**Figure 2:**
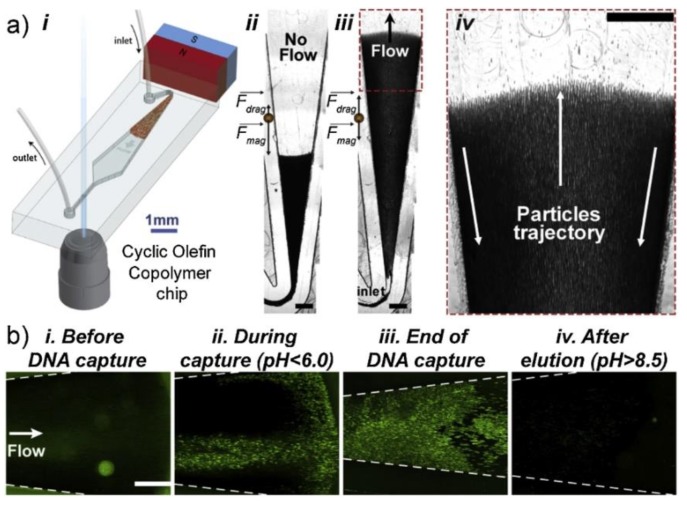
Schematic of the microfluidic Magnetic ExTRactiOn procedure (METRO) using the fluidized bed (**a-i**). Micrographs of the fluidized bed before (**a-ii**) and during (cfDNA) sample added to chip. (**a-iv**) showing the recirculation of beads. Fluorescence micrographs showing DNA-beads interaction inside the fluidized bed, before (**b-i**), during (**b-ii**), at the end of DNA capture step (**b-iii**), and after DNA elution (**b-iv**). Scale bars: 500 μm. Reproduced with permission from [39].

Magnetic bead-based method is another alternative way for DNA extraction. These silica-coated beads have an advantage of effectively removing immobilization step during device preparation. Usually functionalized superparamagnetic beads are mixed and incubated with sample mixture in the chip. On-chip washing is performed with an external magnetic field. As elution buffer comes into the channel, the removal of magnetic field allows DNA-absorbed beads to mix with buffer. Finally, beads are captured by electromagnetic field and eluted DNA is obtained [36]. Magnetic beads-based DNA isolation is widely used to enrich small amount of samples. Verbruggen et al. demonstrated an approach which combined magnetic beads with asymmetric droplet [37]. Such DNA extraction method in a segmented flow microfluidic system successfully removed 90% or even 95% of the original sample volume. More recently, magnetic bead-based approach has been applied to cfDNA isolation (Figure 2), with a constant position of external magnetic field and without the need for an incubation step, which had comparable efficiencies with standard column-based method [38,39]. The emergence of new technology, like 3D-printing, makes magnetic bead-based microdevices more diversified [40].

LOC devices which rely on functionalized surface are also important parts in DNA solid phase isolation. As was reported, there were many ways to generate amine surfaces such as coating microchannel with 3-aminopropyltrietboxysilane (APTES) or 3-[2-(2-aminoethylamino)-ethylamino]-propyluimethoxysilane (AEEA) [41]. The results showed that capacities of isolating DNA in AEEA modified chip were twice as good as the APTES ones. However, the recovery efficiency of DNA extracted from whole blood was only 27–40%, indicating that microchip with amino-coated surfaces had powerless absorption force and might attract other negative species [42]. Photactivated polycarbonate surface is another commonly used method [43,44]. Witek et al. described a microfluidic device with UV-modified surface [45]. With different composition of the immobilization buffer, DNA with different lengths could be concentrated. Considering high fragmentation of cfDNA, the device is promising in cfDNA isolation with high efficiency. More recently, polydimethylsiloxane (PDMS) device with silanes mixture coated surface was proved to achieve much better performance in miR-21 capture and elution compared to the non-functionalized chip [46], which showed the huge potential of functionalized LOC in separation of trace nucleic acids.

The methods mentioned above are all conventional microfluidic DNA isolation ways which can be further improved to be more suitable for cfDNA isolation. However, the length of cfDNA (about 167 bp) might hinder the efficiency of isolation with an increase of sample loss [15]. The yield of cfDNA can also be affected by contamination of high molecule weight DNA fragments resulted from rupture of monocytes [47]. Thus, increasing absorption ability of short fragments is demanded to meet the requirements of efficient cfDNA isolation. Microfluidic devices designed especially for cfDNA isolation have been presented although there are few reports about this issue. Jin et al. [48] demonstrated a dimethyl dithiobispropionimidate (DTBP)-based microchannel platform. They modified the inner surface of channel with 3-aminopropyl diethoxymethylsilane (APDMS). After blood plasma sample was mixed with DTBP and injected into the channel, DTBP bound the amine group of APDMS and cfDNA by covalent bonding and electrostatic coupling. Finally, cfDNA was eluted with breaking the crosslinking by elution buffer. This method provides an opportunity to capture cfDNA within 15 minutes with minimal cellular background. Campos et al. [49], the same group as *Ref.* 45, further developed the device which could be activated with UV/O_3_ to generate carboxylic groups and applied the chip to cfDNA isolation. Immobilization buffer (IB) containing polyethylene glycol and salts triggered cfDNA to be absorbed onto the activated surface. The recovery of cfDNA was >90% and the device also had the ability to isolate short cfDNA fragments (50 bp, >70%), which was superior to commercial kits. Researches related to new polymers and the improvement of reagents benefit the binding of cfDNA to the chip surface, making solid phase isolation have widely application prospects.

#### 2.2.2. Liquid Phase Isolation

**Figure 3:**
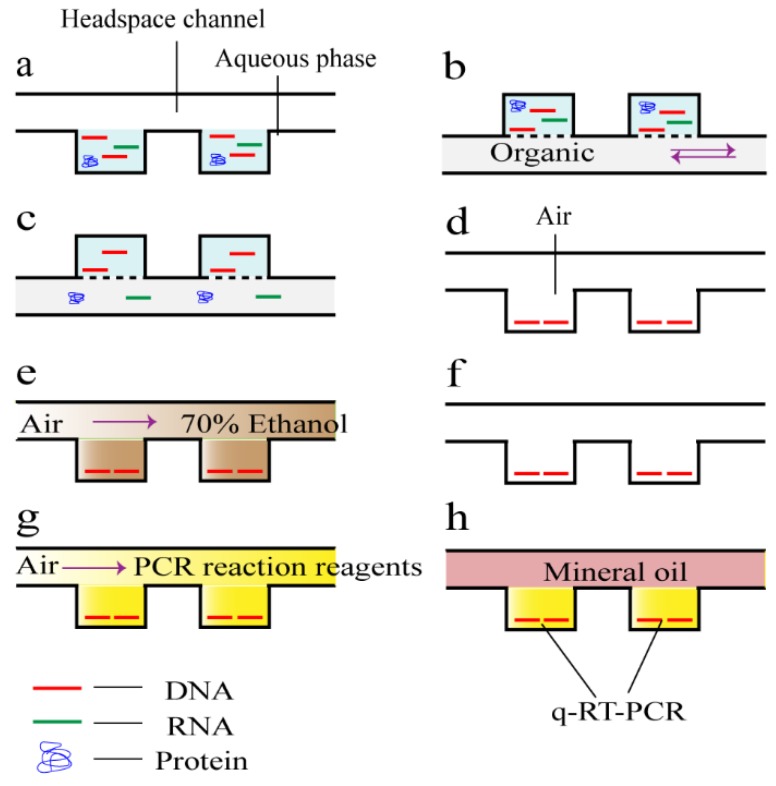
Schematic of the DNA purification process in a liquid-phase DNA isolation chip described by Zhang et al. [51]. (**a**) An aqueous phase containing DNA, RNA, and protein was loaded in the microwells. (**b**) An organic phase with pH 8.0 was introduced into the headspace channel with continuous forward and reverse flow. The chip was inverted for nucleic acid purification in this step as the organic phase has greater density than the aqueous phase. (**c**) Protein and RNA were transferred from the aqueous phase into the organic phase, while DNA remained in the aqueous phase. (**d**) The organic phase was expelled from the headspace channel and evaporated under vacuum while purified DNA in the microwell was concurrently dried (**e**, **f**). Residual organic phase was further decontaminated by repetitive washing and vacuum evaporating with 70% ethanol. (**g**) q-RT-PCR reaction mixture was loaded into the microwells. (**h**) Microwells were covered with mineral oil followed by on-chip q-RT-PCR amplification.

Liquid phase isolation captures DNA with an electric field or chemical reagents instead of functionalized surface or immobilized beads. The chemical reagents approach usually refers to phenol chloroform extracting method, which uses hydroxybenzene-chloroform-isoamyl alcohol (PCI) as the organic phase. When lysate is mixed with PCI, denatured proteins transfer into the organic phase and charged DNA remains in the aqueous phase. Morales et al. [50] reported a droplet-based platform to mix the two immiscible phases with a high recovery yield of >92%. The droplet method improved protein movement from the aqueous phase to the organic one because of the increase of interfacial area and convective enhancement in each droplet. However, the residual organic phase could not be eliminated and influenced downstream analysis. Zhang et al. [51] presented another chip with a 900 microwell array of 125 nL/well where continuous flow of the organic phase increased the contact with the aqueous phase, obtaining 10 fold higher yield compared to commercial kits using column-based solid phase extraction (Figure 3). The residual organic phase evaporated under vacuum, and then removed with 70% ethanol. In general, both the two kinds of device divide large volume of sample into smaller unit, which could largely decrease the required sample volume and make high-throughput isolation and downstream analysis possible. Liquid phase isolation in connection with special chemical reagents achieves high recovery yield but remains reagent residue problem.

Both EP and DEP microdevices have been applied to DNA isolation. EP represents the phenomenon of charged molecules moving towards the opposite electrode under the direct current (DC) electric field while DEP does not require the particle to be charged. DEP refers to a phenomenon where a non-uniform electric field polarizes the particles and exert a force along the field lines on them. As early as ten years ago, gel-electrophoretic-based microfluidic approach was developed for low molecular weight RNA extraction [52]. The device consisted of three parts: sample chamber, gel region filled with crosslinked gel and elution chamber filled with 1× TBE buffer. With a DC constant-current of typically 160 mA, RNA can be extracted within 11 min. The device might be an applicable method for cfDNA. Montes et al. [53] reported a microfluidic device where both convective flow and electrophoretic flow exist. DNA was trapped near the inlet of the channel under the coaction of the pressure gradient and the opposing electric field. Nevertheless, it was stated that there was an upper limit to the amount of DNA in the high concentration region, which hindered large amount of accumulation to some extent. 

**Figure 4:**
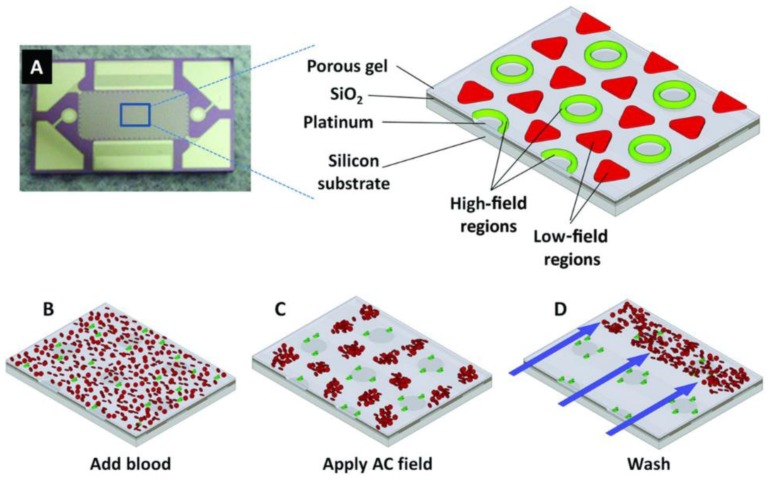
The DEP-based microarray device and scheme for isolation of cfDNA from blood. Reproduced with permission from [55]. (**A**) AC electrokinetic microarray device used with an expanded view of the device materials composition. (**B**) Microarray with whole blood (red) containing fluorescent DNA (green). (**C**) Application of the AC electric field causing the fluorescent DNA (green) to be concentrated in the DEP high-field regions on the microelectrodes, while the blood cells (red) move into the DEP low-field regions between the microelectrodes. (**D**) A fluidic wash removes the blood cells from the microarray under the AC field while DNA remains concentrated in the DEP high-field regions.

It has been decades since DEP was used for biological research in microelectronic chips [54]. Sonnenberg et al. [55] described a DEP-based device to isolate cfDNA from 25 μL unprocessed blood of 15 chronic lymphocytic leukemia (CLL) patients and 3 healthy individuals with a complete processing time of less than 10 min for each sample (Figure 4). The chip was divided into DEP high-field regions (over the microelectrodes) with 1000 microelectrodes and DEP low-field regions (between the microelectrodes). cfDNA could be concentrated into the DEP high-field regions since it was more polarizable than other contaminants at a specific alternating current (AC) frequency and voltage. Impurities were taken away with a fluid wash. However, it remains limitation such as electrochemical damage of platinum electrodes on DEP devices when it comes to the processing of high conductance fluids including blood, plasma and serum [56]. A further study was conducted towards the two model devices—a parallel wire layout and a planar array. With a parallel wire arrangement, fluorescent beads were isolated effectively under low-conductance conditions (10^−4^ S/m) while the device structures were damaged by high electrothermal flow under high conductance conditions (>0.1 S/m). Planar devices were proved to be effective under high conductance conditions (~1 S/m). cfDNA was successfully isolated from un-diluted plasma using a planar device under 20 min with an AC electric field at 14 Vpp and 15 kHz. While EP/DEP-based approaches provide better purity, the complexity of fabrication and damage of electrodes limit the large scale production and stable operation of chips. Both the solid and liquid phase isolation methods are summarized in Table 1 for comparison.

## 3. Quantification of cfDNA

The absolute concentration of cfDNA is a promising independent biomarker for overall survival (OS) of cancer patients, which could play an important role in cancer diagnosis and treatment. Thus, precise quantification of cfDNA is highly demanded and has attracted many researchers’ concern. Also, rapid quantification is in need for POCT to distinguish the cancer patients. Commonly used approaches for cfDNA quantification include UV spectrophotometry, fluorescent dyes, quantitative PCR (qPCR) and digital PCR (dPCR) [15]. These approaches have been performed on chip for reducing sample loss due to the process of sample transfer and for more accurate quantification (Table 2).

Fluorescent dyes which can bind double stranded DNA specifically are widely used in quantification due to its sensitivity and convenient operation. The fluorescent dyes available like PicoGreen and Qubit assays have a detection accuracy of pg/mL. Quantification of cfDNA with fluorescent dyes in microfluidic devices is usually integrated with EP/DEP-based isolation method (Figure 4) [55]. In this approach, fluorescent dyes are added to samples before isolation. After EP/DEP is carried out and impurities are removed by a fluidic wash, the concentrated cfDNA in a certain region is detected directly on chip and normalized to the original sample volume. By detecting the fluorescence intensity, higher concentration of cfDNA was found in chronic lymphocytic leukemia patients than the healthy samples. Yang et al. [57] adopted the similar approach in combination with gel electrophoresis in channels to distinguish the severe patients from the healthy ones. As fluorescently labelled cfDNA migrated in gel under a DC electric field, fluorescence images were collected at a periodic interval to monitor the fluorescent intensity to determine the concentration of cfDNA. The integrated chip allows isolation and quantification of cfDNA from the whole blood or plasma to be conducted without transfer of samples between centrifuge tubes, which reduces the loss during operating process. In all, direct on-chip optical detection using commercial kits like PicoGreen assay takes the most prominent advantages in rapidness and convenience, which is promising in POCT for cancer diagnosis.

RT-qPCR, which consists of TaqMan and ARMS assays, is the most commonly used way to quantify cfDNA in tubes with large sample volume [65,66], with the limit of detection (LOD) of ~0.02 pg [67]. The TaqMan probe, which contains a fluorophore and a quencher, is complementary to part of PCR product. Primer which is complementary to the mutant site allows PCR reaction to extension, removing the fluorophore from probe and resulting fluorescence. The assay is conducted by comparing the abundance of housekeeping genes or some stable non-coding repeat sequences like *β-globin*, *GAPDH*, *TERT*, *RPPH1*, *ERV3*, *MSTN* and *ALU* to a standard curve of known concentrations. Hurth and colleagues [58] reported a microfluidic device for RT-qPCR with reaction volume of less than 20 μL. The qPCR microdevice was embedded in an automated system for loading sample and profile-out. With high degree of automation which allows for continuously processing sample, the team determined a DNA concentration of ~1 ng/μL and achieved a precise correlation with the commercial device. RT-qPCR has been used on the same chip where isolation of DNA is performed, for the purpose of decreasing sample loss (Figure 3) [51]. However, microdevices related to RT-qPCR of cfDNA are not widely used as other methods probably because it is more time-consuming than detection directly with fluorescent dyes and less precise than dPCR.

Digital PCR (dPCR), where individual DNA molecules can be dispersed into thousands of smaller units, provides a more sensitive way to determine the absolute level of cfDNA. dPCR allows each unit to either contain one to several molecules or not contain DNA template. After PCR amplification, each unit was detected where the existence or absence of fluorescence signal was recognized as 1 or 0, respectively. According to the number and proportion of positive units, the initial copy number or concentration of target molecule could be obtained by poisson distribution principle. dPCR usually relies on microfluidic chips with numerous reaction wells or droplet method (ddPCR). It takes an advantage in throughput compared to microarrays or qPCR methods due to its ability to detect large number of wells or droplets at the same time. The standard ddPCR process involves the application of microfluidic techniques for generating droplets and droplet fluorescence reading. As for droplet fluorescence reading, there are mainly two strategies available. One is to flow droplets in a single file for read-out using a flow cytometry apparatus, the other is to take image of the chip, followed by powerful image analysis. dPCR or ddPCR has been commercialized as an integrated system and widely used in cfDNA research [61,62]. For example, Chen and colleagues used ddPCR targeting *EFTUD2* and *TRAK2* to determine cfDNA yield of patients with non–small cell lung cancer, obtaining a range from 4 to 8,000 ng and median yield of 59 ng [61,62]. However, the sensitivity of dPCR depends on the number of microwells or droplets, so the ability to create more microchambers or to generate more droplets stably is a future direction for improvement [59,60].

Although PCR performed in microfluidic devices may reduce the over amplification of dominant fragments to some extent, high fragmentation of cfDNA can lower PCR efficiencies. As a result, quantification measured by PCR-based methods including RT-qPCR and dPCR could be underestimated, which remains further development [15].

## 4. Mutation Detection of cfDNA

It has been a consensus that cfDNA can inform genomic mutation [5,6]. Thus, cfDNA helps early detection of cancer and is also a promising way to monitor cancer progression and detect drug-resistant mutations for personalized medicine. 

### 4.1. Commercialized dPCR/ddPCR System and Application

Methods most commonly applied for mutation detection of cfDNA in microfluidic devices are similar to the ways for quantification. While chips based on DNA hybridization for fluorescence readout [68] or on-chip qPCR [51] are also potential methods for cfDNA detection, dPCR/ddPCR is the most widely used microfluidic-based way in cfDNA analysis, with a lower limit of detection (LOD: 0.001%) than traditional ways like ARMS (LOD: 0.05–0.1%) [69]. Due to the “mixture” property of cfDNA, the fraction of DNA derived from tumor cells (ctDNA) which is called tumor fraction should be taken into consideration. As was reported, Diehl and colleagues found that ctDNA/cfDNA in 33 colorectal cancer patients ranged from 0.01% to 1.7% [70]. Similar results were obtained in a study of Azad et al. where they determined a frequency of 0.1–23% (median 1.5%) targeting exon 8 of the AR gene in cfDNA of metastatic castrate resistant prostate cancer patients [71]. Low frequency of mutation makes it difficult to identify tumor-derived cfDNA from large background, and dPCR/ddPCR provides a good solution for detection targeting specific gene.

Microfluidic chips for dPCR and ddPCR are available on sell and have been integrated as a complete system. The sensitivity and throughput of detection rely on the number of wells or droplets. Commercially, the commonly used QuantStudio 3D Digital PCR System (Applied Biosystem) contains 20,000 independent reaction wells in each chip. For ddPCR, the QX200 Droplet dPCR System could generate 20,000 nanoliter-sized droplets while the Naica Crystal Digital PCR System (Stilla Technologies) could partition sample into 30,000 droplets. The ability has been further developed to generate 10 million picoliter-sized droplets with the RainDrop Plus Digital PCR System (RainDance Technologies). dPCR or ddPCR system has been widely applied in cfDNA research for mutation detection and quantification. Kasahara et al. [72] used commercialized dPCR system to detect 3 *EGFR* mutations (exon 19 deletions, L858R in exon 21, and T790M in exon 20) in cfDNA of patients with advanced non-small cell lung cancer, and LOD of each assay was determined to be 0.1% in this study. Farkkila et al. [73] detected the FOXL2 402C>G (C134W) mutation in cfDNA of patients with adult granulosa cell tumors using a ddPCR assay with a low sensitivity of 0.05%. Other “hot spots” like *ESR1*, *BRAF*, *KRAS* and so on have been selected for liquid biopsies using ddPCR [6,74]. While the sensitivity has small differences between different spots, dPCR/ddPCR is a powerful tool for clinical diagnosis.

### 4.2. Further Developed Methodological Study for Detection

**Figure 5:**
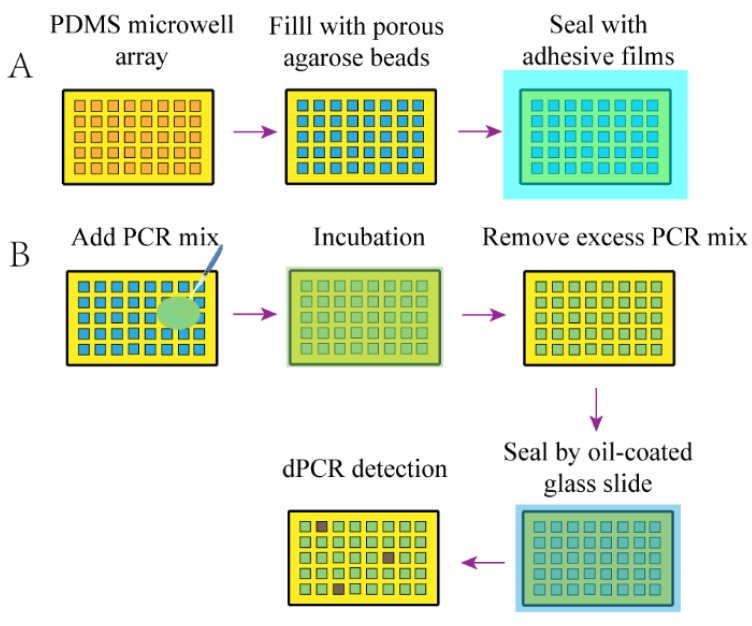
dPCR using a superporous absorbent array chip [77]. (**A**) Schematic of fabrication of the ready-to –use microdevice. Dry agarose microparticles are packed in a PDMS microwell array. (**B**) Workflow of on-chip dPCR. PCR mix with DNA is added and covers the surface of the chip. After incubation for microbeads to fully absorb the sample and reagents, excess PCR mix is removed. A glass slide with fluorocarbon oil seals the chip to isolate each microwell. Finally, dPCR is conducted in a thermal cycler, followed by fluorescence detection.

Microfluidic chips with nanoliter and even picoliter well array have been used for DNA analysis for a long time [75]. For further facilitating dPCR in clinical practice, Zhu et al. [76] utilized high gas solubility of PDMS to provide surface tension for sample spontaneous compartmentalization, realizing parallel detection of 480 to 4804 template molecules on one panel with 5120 microchambers of 5 nL. This SPC named chip for dPCR with high-throughput benefited automation and integration without complex pipeline. Wang et al. [77] achieved the similar goal that the microchambers in array could be spontaneously filled using a superporous absorbent array chip (Figure 5). The chip packed with microbeads performed superior ability for sample absorption. With an array of 50 × 50 microwells, they achieved comparable results with ddPCR. The two devices are mainly focused on equipment-free sample partitioning, which is a direction for improvement to satisfy the need of total automation.

**Figure 6:**
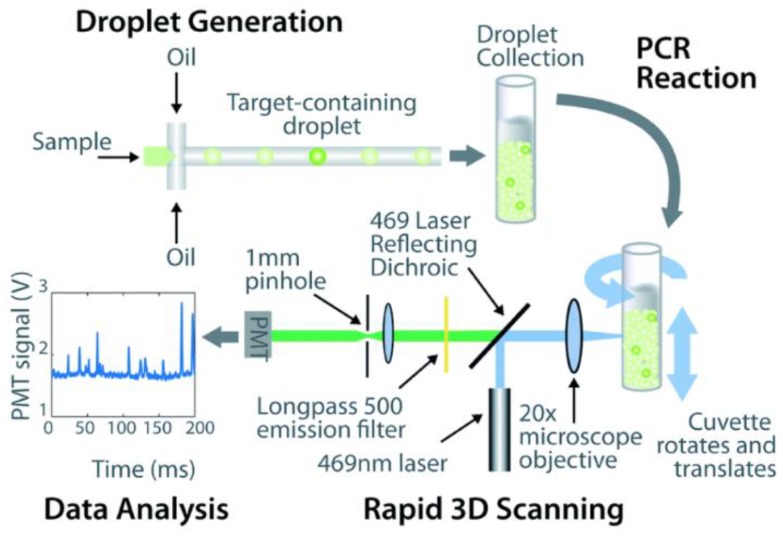
Workflow of IC3D ddPCR for clinical samples. Reproduced with permission from [78]. Sample is divided into millions of picoliter-sized droplets. After PCR amplification, target fluorescent droplets are transported across the excitation volume due to the slow vertical translation and fast rotation of the cuvette and finally quantified.

Efforts in developing ddPCR for cfDNA mutation detection to achieve an ultrasensitive test have been reported, probably because droplet-based method is much easier to adjust the number of droplets compared to fabrication of microwell array with different scales. Thus, it seems to be more suitable to improve the cfDNA detection sensitivity based on ddPCR. Ou and colleagues [78] described an integrated comprehensive droplet digital detection (IC3D) digital PCR system (Figure 6). The system combined target-specific fluorescent chemistry, droplet microfluidics and a high-throughput 3D particle counting technology. The system took advantages in the ability to analyze larger sample volumes with greater number of partitions compared to commercial dPCR system, increasing sensitivity consequently. Using spike-in analysis, KRAS G12D mutant alleles were detected at a sensitivity of 0.00125–0.005% with a false positive rate of 0%, which is 50 to 1000× more sensitive than existing commercial ddPCR and qPCR platforms, respectively.

Microfluidic channels have been combined with biosensing technologies for on-chip detection of cfDNA fragments [79]. Probes consist of oligonucleotides have been used for capturing cfDNA fragments with specific sequences. Dias et al. [63] presented a strategy of a biochip platform integrating 30 magnetoresistive (MR) sensors and a microfluidic channel for cfDNA cancer diagnostics. For concept proving, the target fragments with ALU repetitive sequence were chosen and amplified, followed by single-strand targets generation and labelling with magnetic nanoparticles. After magnetically labeled fragments were introduced into the microfluidic channel, the targets could be hybridized with probes immobilized on the gold substrates. MR sensors were used for signal readout to assess the DNA integrity and quantify ALU elements within the picomolar range. On-chip interrogation of single-nucleotide polymorphisms (SNPs) on cancer-derived cfDNA has been developed using electrochemical sensors [64]. Such sensor could be integrated in a microfluidic device for rapid detection and for an entire manipulation of cfDNA molecules from extraction to mutation detection without liquid transfer.

Generally, the mainstream method of mutation analysis with microfluidic technologies is PCR-based with fluorescence for optical detection since PCR makes cfDNA of low abundance to be amplified, which lowers the limit and introduces PCR efficiency problem at the same time. dPCR in microwell array or ddPCR could be conducted for high-throughput mutation screening and absolute quantification of mutation. Nonetheless, the number of microwells or droplets sets up a preset capacity for sensitivity and quantification range.

## 5. Library Construction for Next-generation Sequencing

Since mutation detection by one actionable alteration makes cancer diagnosis less accurate, combination of several assays targeting multiple mutant alleles using different dyes was developed in a single experiment. The assay becomes bloated with the involvement of more targeted genes although a relatively small panel could achieve high sensitivity and specificity. Detection of copy number aberrations (CNA) is still challenging for smaller targeted panels since it is difficult to determine the mutation from large background [3]. However, such detection needs prior knowledge of the certain mutations and only a portion of patients carrying the mutation can be targeted, which hinders its application in clinical diagnosis. 

With the development of read length and quality as well as the reduction of cost, next-generation sequencing (NGS) provides more opportunities for cfDNA analysis in addition to unknown mutation detection. For example, tumor fraction can be predicted using bioinformatics methods to analyze sequencing data [13]. Unlike traditional methods where fragments with specific mutation are regarded as tumor-derived DNA, NGS helps utilize the entire data to perform the prediction. Chen et al. [62] reported that tumor fraction ≥10% was prognostic for overall survival using whole-genome sequencing data of cfDNA, which shows great potentials for cfDNA analysis relying on NGS.

**Figure 7:**
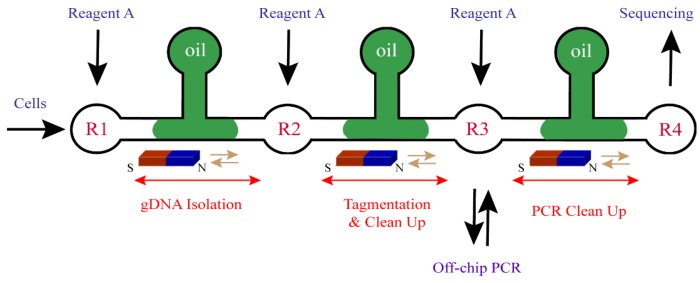
Schematic of microfluidic workflow [81]. Water or aqueous buffer exists in each reservoir (R1–R4). Cells are added in R1 and incubated, followed by adding of reagent A (magnetic beads and binding buffer). The beads binding with DNA are moved to R2 and then DNA is eluted from the beads in reaction buffer. DNA fragmentation and adapter ligation reaction are performed in R2. After reagent A is added and beads are moved to R3, DNA is eluted from the beads into water. DNA is transferred for off-chip PCR amplification and then moved back to R3. With Reagent A added in R3 and beads moved to R4, purified DNA is prepared for sequencing.

The process of traditional sequencing library construction for NGS consists of end repair, adding A-tail, ligating adaptor and library amplification. Although a number of commercial kits have launched to increase efficiencies and reduce operating time, the process is still considered time-consuming and labor-intensive with many steps to transfer and purify samples, which results in sample loss and has a chance of contamination. Although sequencing library construction of single cell has been conducted in microfluidic devices in combination with Tn5 transposase for high throughput analysis of environmental microorganisms [80], microfluidic device designed for cfDNA is still limited. Snider et al. [81] presented a sample preparation workflow of human genomic DNA for NGS in microfluidic channels, which achieves comparable sequencing results with available NGS library preparation kit optimized for low-input samples (Figure 7). In this method, four aqueous reservoirs with buffer were separated by three regions of oil filled channels. A single step was performed in each reservoir. Magnetic beads were used to bond DNA and control its motion between the two phases. Although the PCR amplification step of adaptor-ligated fragments in this approach was finished out of chip, it provides an inspiration for us of the microfluidic device targeting cfDNA. Kim et al. [82] described a fully-integrated and more automated platform to prepare sequencing library for clinical sample which had limited quantity and required precise handling at small scale. It was suitable for cfDNA and had the ability to deal with samples in large numbers. The system used a digital microfluidics (DMF) hub to integrate several reagent and sample manipulation modules which involved magnets for size-selection as well as clean-up and thermal blocks for tagmentation and PCR. The DMF hub relied on indium tin oxide (ITO) actuation electrodes to force droplets to move between each module and to fuse with the droplet of reagents. Such platform could be developed as a potential way for cfDNA library preparation for NGS.

The microchip designed especially for cfDNA sample preparation could omit the fragmentation step, which makes the device less complex. Since PCR bias and errors could be introduced during the sequencing library construction process, unique molecule identifiers (UMI), which can label single molecule, have been widely applied for removing PCR duplication in the downstream analysis and reducing false positive rate. UMI can be introduced during the process of sequencing library preparation in a microchip because it is convenient to implement using droplet microfluidics [83]. 

Although we reviewed the microfluidic methods applied to cfDNA research by four independent steps, actually the microfluidic platform described above may involve more than one function in a chip. The integration of multiple functions is also a main advantage that LOCs bring [46], which makes cfDNA clinical research more convenient and efficient. For example, the device mentioned in *Ref.* 51 allows for DNA capture and RT-qPCR for quantification or mutation detection (Figure 3). Devices introduced in *Ref.* 54 (Figure 4) and *Ref.* 57 contain the function of enrichment and quantification while chip in *Ref.* 81 (Figure 7) can realize the functions of isolation and sequencing library preparation. More efforts could be made to integrate more functions in one chip. For example, after collecting cfDNA from blood, the entire DNA could be divided into several parts and flow to different channels for individual downstream analysis.

## 6. Conclusions and Future Perspectives

cfDNA, as a liquid biopsy for cancer, has drawn extensive attention for its abilities to inform genomic mutations, tumor burden and potential mechanisms of drug resistance. However, its properties of low abundance and high fragmentation hinder precise quantification and further analysis. Also, the traditional ways are time-consuming, which limits the POCT of cfDNA. The development of microfluidic devices provides a more rapid way for cfDNA isolation with smaller required sample volume and make accurate quantification and high-throughput screening possible. Furthermore, it could integrate and automate the whole cfDNA detection process from isolation to analysis.

Microfluidic techniques related to cfDNA is limited and have not been widely explored. As for cfDNA capture and enrichment, the potential isolation devices are classified as solid phase isolation and liquid phase isolation methods. Both silica pillars-based and silica particles-based methods for DNA solid isolation have comparable efficiencies with standard column-based method with a smaller required sample volume. These potential methods should be further improved for cfDNA isolation in terms of buffer selection and channel design to enhance non-specific absorption of cfDNA with high surface-area-to-volume ratios. A systematic comparison needs to be conducted between these microfluidic devices to analyze the efficiency of each method. The microfluidic devices designed especially for cfDNA in recent years utilizing covalent bonding or electrostatic coupling with polymeric materials showed high efficiencies which were superior to commercial kits. Liquid phase isolation using PCI achieves high recovery yield but remains reagent residue problem. EP/DEP-based methods provide better purity while the manufacturing process of chips is complex and electrodes tend to be electrochemically damaged after a period of operation. The appropriate method can be selected according to the requirements of downstream analysis. Both the solid and liquid phase extraction methods could separate DNA sample into subunits by means of silica pillar array or microchamber array structure for the aim of high-throughput isolation and integration with downstream analysis.

Quantification plays an important role in cfDNA analysis. Approaches including fluorescent dyes, qPCR and dPCR/ddPCR have been performed on chip for reducing sample loss due to liquid transfer. The use of fluorescent dyes-labeled cfDNA makes it easier for direct quantification. Combined with EP/DEP-based isolation method, the integrated chip realizes the rapid detection of cfDNA with lower input. dPCR/ddPCR allows for separating cfDNA molecules into numerous micro reaction wells or droplets for high-throughput screening of these small units to achieve more accurate quantification while high fragmentation of cfDNA may decrease PCR efficiencies and make results underestimate. Efforts could be made in cfDNA quantification to lower the detection limit with non-PCR methods and to compare results of microfluidic PCR-based and PCR-free methods using fragmented DNA with defined concentration. 

Approaches to mutation detection in micro channels are similar to those of quantification. dPCR/ddPCR is able to increase throughput and sensitivity, which is determined by the number of microwells and droplets. Further efforts should be taken to make breakthrough in microwell array chip fabrication, droplet generation and detection, or in sample self-compartmentation to realize automation. The combination with biosensing technologies bring about new opportunities for cfDNA analysis. MR sensors and electrochemical sensors-based methods have been used for detection with a high sensitivity. 

NGS is another method widely used since it provides more information and is easier to detect CNA from large background. Sequencing library preparation in microfluidic devices decreases sample loss, obtaining comparable sequencing results with available NGS library preparation kit optimized for low-input samples. Improvements could be undertaken on such device including integrating on-chip PCR amplification step and applying UMI, for analyzing samples with a small amount of tumor-derived cfDNA. The isolation step also could be integrated to make the assay more convenient and time-saving. 

Future development may focus on more integrated LOCs and provide clinical standards for multiple cfDNA analysis. The ease that microfluidic technologies bring will make cfDNA analysis more widely used in clinic as a liquid biopsy.

## Figures and Tables

**Table 1:** Summary of cfDNA isolation techniques.

Isolation Method	Description of Method	Sample	Volume	Capture Efficiency or Results	Isolation Time	Ref.
Commercial kits	Column-based or magnetic bead-based	Plasma, serum, urine	>1 mL	50–100%	>60 min	[25]
Solid phase isolation	Dynamic magnetic extraction	Serum	30–60 μL	64 ± 9%	~2.5 h	[39]
	DTBP-based microchannel platform	Plasma	~200 μL	Similar to the input as an absolute value	<15 min	[48]
	Chip activated by UV/O_3_	Plasma	7–717 ng	>90% for 100–700 bp, >70% for 50 bp	-	[49]
Liquid phase isolation	DEP-based device	Unprocessed blood	25 μL	Comparable to commercial kits	<10 min	[55]
	DEP-based planar device	Plasma	25 μL	-	<20 min	[56]
	EP-based device with gel	Plasma	<10 μL	-	~5 min	[57]

**Table 2:** Summary of microfluidic techniques for cfDNA quantification and mutation detection.

Approach	Advantage	Ref.
Quantification		
On-chip direct optical detection	Easy to integrate for less sample loss, simple-structured, rapid, real-time monitoring	[55,57]
On-chip RT-qPCR	Integrated with isolation process to reduce sample loss, simple-structured, more sensitive, real-time monitoring, automated	[51,58]
dPCR/ddPCR	Most sensitive, precise quantification to single molecule, automated, high-throughput	[59,60,61,62]
Mutation detection ^1^		
Biochip platform with MR sensors	High sensitivity within picomolar range and increased portability, greater ability to discriminate homologous or truncated sequences	[63]
Electrochemical-based chip	Sensitive, stable, reusable, no need of adding exogenous reagents, rapid (minutes)	[64]

^1^ The three quantification approaches list above are also potential methods for mutation detection.

## Abbreviations

DNA	deoxyribonucleic acid
PCR	polymerase chain reaction
LOC	lab-on-a-chip
cfDNA	cell free DNA
NIPT	Non-Invasive Prenatal Testing
POCT	point-of-care testing
EP	electrophoresis
DEP	dielectrophoresis
APTES	3-aminopropyltrietboxysilane
AEEA	3-[2-(2-aminoethylamino)-ethylamino]-propyluimethoxysilane
PDMS	polydimethylsiloxane
DTBP	dithiobispropionimidate
APDMS	3-aminopropyl diethoxymethylsilane
PCI	hydroxybenzene-chloroform-isoamyl alcohol
DC	direct current
CLL	chronic lymphocytic leukemia
AC	alternating current
OS	overall survival
qPCR	quantitative PCR
dPCR	digital PCR
ddPCR	droplet digital PCR
LOD	limit of detection
MR	magnetoresistive
SNPs	single-nucleotide polymorphisms
CNA	copy number aberrations
UMI	unique molecule identifiers
NGS	next-generation sequencing
DMF	digital microfluidics
ITO	indium tin oxide
